# Registration of retinal sequences from new video-ophthalmoscopic camera

**DOI:** 10.1186/s12938-016-0191-0

**Published:** 2016-05-20

**Authors:** Radim Kolar, Ralf. P. Tornow, Jan Odstrcilik, Ivana Liberdova

**Affiliations:** Department of Biomedical Engineering, Faculty of Electrical Engineering and Communication, Brno University of Technology, Technicka 12, 616 00 Brno, Czech Republic; Department of Ophthalmology, Friedrich-Alexander-University Erlangen–Nürnberg, Schwabachanlage 6, 91054 Erlangen, Germany

**Keywords:** Retinal imaging, Video-ophthalmoscopy, Image registration, Tracking

## Abstract

**Background:**

Analysis of fast temporal changes on retinas has become an important part of diagnostic video-ophthalmology. It enables investigation of the hemodynamic processes in retinal tissue, e.g. blood-vessel diameter changes as a result of blood-pressure variation, spontaneous venous pulsation influenced by intracranial-intraocular pressure difference, blood-volume changes as a result of changes in light reflection from retinal tissue, and blood flow using laser speckle contrast imaging. For such applications, image registration of the recorded sequence must be performed.

**Methods:**

Here we use a new non-mydriatic video-ophthalmoscope for simple and fast acquisition of low SNR retinal sequences. We introduce a novel, two-step approach for fast image registration. The phase correlation in the first stage removes large eye movements. Lucas-Kanade tracking in the second stage removes small eye movements. We propose robust adaptive selection of the tracking points, which is the most important part of tracking-based approaches. We also describe a method for quantitative evaluation of the registration results, based on vascular tree intensity profiles.

**Results:**

The achieved registration error evaluated on 23 sequences (5840 frames) is 0.78 ± 0.67 pixels inside the optic disc and 1.39 ± 0.63 pixels outside the optic disc. We compared the results with the commonly used approaches based on Lucas-Kanade tracking and scale-invariant feature transform, which achieved worse results.

**Conclusion:**

The proposed method can efficiently correct particular frames of retinal sequences for shift and rotation. The registration results for each frame (shift in *X* and *Y* direction and eye rotation) can also be used for eye-movement evaluation during single-spot fixation tasks.

## Background

Retinal imaging is an important part of ophthalmology and is also used in other research areas such as neurology and cardiology because it makes it possible to image retinal vascularity and retinal nerve fibres. Most light-based retinal imaging modalities can acquire static images of the retinal tissue—e.g. fundus camera, optical coherence tomography (OCT) or scanning laser ophthalmoscopy (SLO)—thus enabling assessment of the retinal structures. Some modalities (e.g. laser speckle flowmetry or Doppler OCT) can also measure blood flow through the retinal vascular tree, which is connected with functional assessment, particularly when specific light stimulation is used. However, there is a lack of low-cost devices that can measure fast retinal dynamic changes under low-light conditions, i.e. non-mydriatically. The only two commercially available devices are mydriatic and relatively expensive. The first one, based on laser speckle contrast imaging (LSFG-NAVI, Softcare, Japan), is capable of measuring relative changes of blood flow under coherent illumination. The second device is a retinal vessel analyser (DVAplus, Imedos, Germany), which analyses blood vessel diameter and pulsation. Here, we briefly describe our low-cost experimental video-ophthalmoscope (VO) with non-coherent light source [1], which is capable of acquiring long retinal video sequences in non-mydriatic eyes with high spatial and temporal resolution. The main part of this paper describes a method for registering acquired retinal video sequences, which is a necessary part of the imaging system because it performs an offline *image stabilisation.* Possible applications of a registered sequence are: analysis of the blood-vessel pulsations (fast calibre changes during cardiac cycle), eye-movement estimation (directly from estimated parameters of geometrical transform during the registration process), or creation of an averaged retinal image with higher image quality (with respect to noise and resolution, when deconvolution is used).

There are many published methods for retinal image registration of static images, either mono or multimodal. They are usually based on landmark correspondence as the retinal vascularity is considered a source of reliable landmarks [2]. Some papers also describe intensity-based registration [3, 4], their combinations [5], or combination with other approaches [6, 7]. Nevertheless, retinal image registration is still challenging in some applications or modalities. Our experimental VO acquires sequences under low illumination, resulting in more noisy images compared with images from current colour fundus cameras. The retina can also move very fast and substantially during acquisition, due to (micro) saccadic eye movements. Therefore the registration approach must be robust against the noise and artefacts caused by eye movements as well as eye blinking. Video sequences have, typically, a few hundred frames. Therefore, the registration process must be fast in order to align the sequence in a reasonable amount of time on common hardware (tens of seconds can be acceptable for clinical usage). These specific features and needs make the registration process a challenging task. There are only a few papers so far that are close to our application (i.e. fast registration of retinal temporal sequences acquired from a fundus camera). Now, we will discuss these papers and also other state-of-the-art papers presenting a similar registration approach.

Perez-Rovira et al. [8] described a method for registering high-resolution, ultra-wide field-of-view retinal sequences acquired during fluorescence angiography. Their approach is based on blood-vessel tree segmentation and bifurcation points matching, using a pairwise registration of whole sequence taking one frame as a *reference* and the other frames as *moving*. Their sequences contain between 8 and 27 frames with relatively low levels of noise due to the use of a commercial retinal camera (Optos P200C). They claimed 96.4 % correctly registered frames (graded subjectively by a physician).

Scharcanski et al. [9] used infrared retinal images acquired under 940 nm illumination with framerate equal to 13 fps. They detected significant eye/retinal motion using a metric based on mutual information. The movement estimation was performed using the Fourier shift theorem. The authors reported a pixel difference error (normalised difference between two frames) of 0.065 %, evaluated on 2710 frames. Although the evaluation measure is lower in comparison to the other two techniques that they used for comparison (Lucas-Kanade tracking with scale-invariant feature transform (SIFT) feature and correlation of magnitude), it doesn’t offer straightforward information about the registration accuracy.

An increasing number of applications of image registration can be found in the area of retinal imaging with adaptive optics (AO). However, these applications mainly use a scanning acquisition process, making the registration task different from ours. Nevertheless, Ramaswamy et al. [10] described a successful application of the phase correlation method for elimination of residual fundus image motion (shift and rotation) acquired *by non*-*scanning rtx1* retinal device (Imagine Eyes, France). They observed that the phase-correlation approach could achieve promising results with respect to retinal nerve fibre sharpness measured by texture analysis. The same research group [11] also published an approach for registering AO-corrected retinal images from the same device, which uses a cross-correlation technique and least-squares, and a shape-preserving Procrustes algorithm to determine translation, scaling and rotation. The evaluation is based on the sharpness of the cone contrast in an averaged image, which doesn’t quantitatively evaluate the precision of the registration method. Sequences from the same device have been used in Kulcsar et al. [12] for analysis of local motion. The authors used the iterative Lucas-Kanade approach for estimating residual blood-vessel motion activity. Li et al. [13] describe the application of Lucas-Kanade tracking for processing of 1° field-of-view sequences from AO confocal SLO, providing real-time 30 fps retinal imaging. The authors used the SIFT approach for stable feature detection. Finally, second-order polynomial geometric transformation is used for frame alignment. Unfortunately, the authors didn’t provide quantitative evaluation of registration results.

From the above-mentioned papers it can be concluded that the application of phase correlation in retinal imaging is rather popular and still increasing because of its efficiency and possibility of fast implementation. Other popular approaches use (usually) SIFT features, which are tracked by the Lucas-Kanade algorithm. In this paper, we adopt these two approaches for registration of our video sequences. The presented method is a continuation of our previous work [1, 7, 14], in which we applied these approaches on standard fundus images as well as colour and grayscale retinal video sequences. Nevertheless, there are still some issues that have to be addressed. The first one is the selection of the reference frame, which is an important task for successful registration. The frame should have high contrast with the blood vessels without blur or reflection. The second issue is the robustness of the method to relatively high levels of noise, blur and reflection artefacts. The third important part of the registration method is reliable and quantitative evaluation of the results, which should be objective and easy to perform.

The paper is organised as follows. We describe the data acquisition in “Methods” section. The detailed description of the proposed registration approach is presented in “Results” section, followed by evaluation and discussion in “Conclusion” section.

## Methods

### Data acquisition

The image sequences were acquired at the Department of Ophthalmology, University of Erlangen-Nürnberg, Germany, in the framework of the Erlangen Glaucoma Registry (EGR), a clinical registry (ClinicalTrials.gov identifier: NCT00494923). The experimental non-mydriatic VO has been used for acquisition of 23 sequences from healthy subjects. The number of frames in particular sequences varies from 127 to 474, resulting in a total of 5840 frames used for the processing.

The main principle of the experimental VO has been described in Tornow et al. [1]. Here we summarise the main properties. The VO principle is similar to a fundus camera. An ophthalmoscopic lens (40D) forms an intermediate aerial image of the retina in the image plane. This image is reimaged by the system of two achromatic lenses (120 mm focal length) to the camera sensor. The field-of-view is 20° × 15° with the image centred on the optic nerve head (ONH). Compared with the fundus camera, the main difference is the illumination pathway—we use the central area of the pupil for illumination (entrance pupil) and the rest of the pupil is used for imaging (exit pupil), similarly as used in SLO. A simple light-emitting diode at 575 nm is used as a light source, which is placed in the conjugate plane of the pupil centred on the optical axis. Due to pupil magnification in the system, the image of LED in the pupil plane of the subject is reduced to 1 mm. A CCD camera (UI-2210 SE-M-GL, 640 × 480 pixels, USB interface, iDS, Germany) is used as image detector with 25 fps to take short video sequences. One image pixel corresponds to 9.3 µm on the retina and a visual angle of 1.86 arcmin (for eye axial length of 24.3 mm).

### Image registration overview

**Fig. 1:**
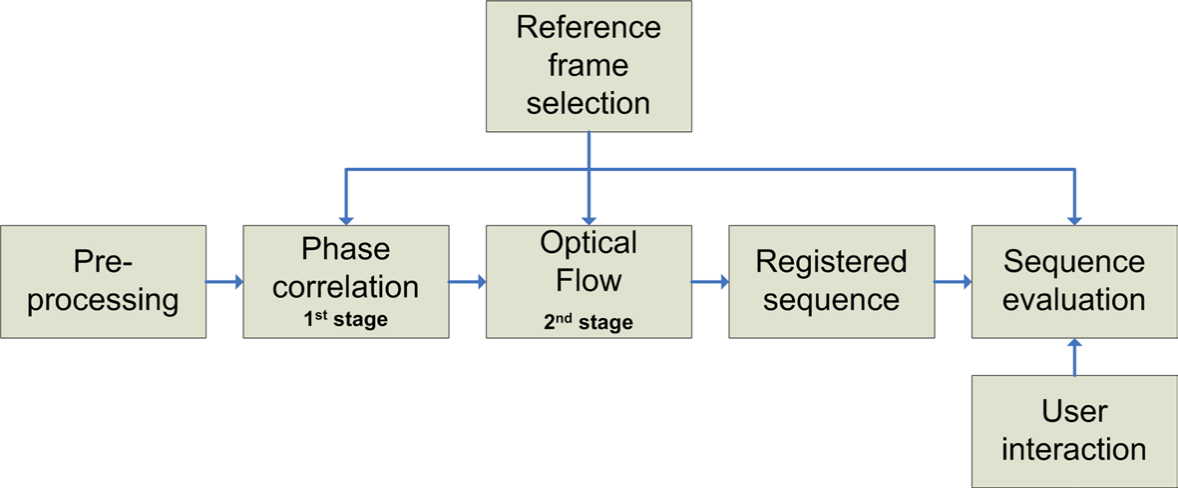
An overview of presented approach for retinal sequence registration and evaluation

The concept of the proposed registration approach is shown in Fig. 1. Our approach is based on one reference frame selected from a sequence. Then, pre-processing must be employed in order to normalise the unequal frame contrast and illumination. As a result, a two-stage registration is used—large eye-movement compensation is followed by fine frame adjustment. The registration evaluation is also discussed as an important part of the image-registration task.

### Reference-frame selection

Selection of the reference frame is a critical step in many image-registration approaches. We use a single reference image approach, i.e. the same reference image is used for registering the whole sequence in both processing stages.

There are two main artefacts, which must be considered when selecting the reference frame and which might cause difficulties in the registration process—*blur* due to eye movements and *lightreflection* due to the eyes blinking and strong corneal reflection. Both artefacts influence the retinal image and corresponding image histogram. A simple and efficient solution for detecting these distorted frames is based on an entropy metric. Each frame *g(x,y)* can be considered as a blurred image of original scene *f(x,y)*, corrupted by an additive noise *n(x,y)*:

$$g(x,y) = f(x,y)*h(x,y) + n(x,y)$$

The point spread function (PSF) *h(x,y)* includes the time and spatial invariant part of the optical-imaging system and also the time-variant distortion caused by eye movements. In order to evaluate blurriness, we can enhance edges using convolution operator *s(x,y)*:

$$l = g*s = f*h*s + n*s = \left( {f*s} \right)*h + n*s$$

**Fig. 2:**
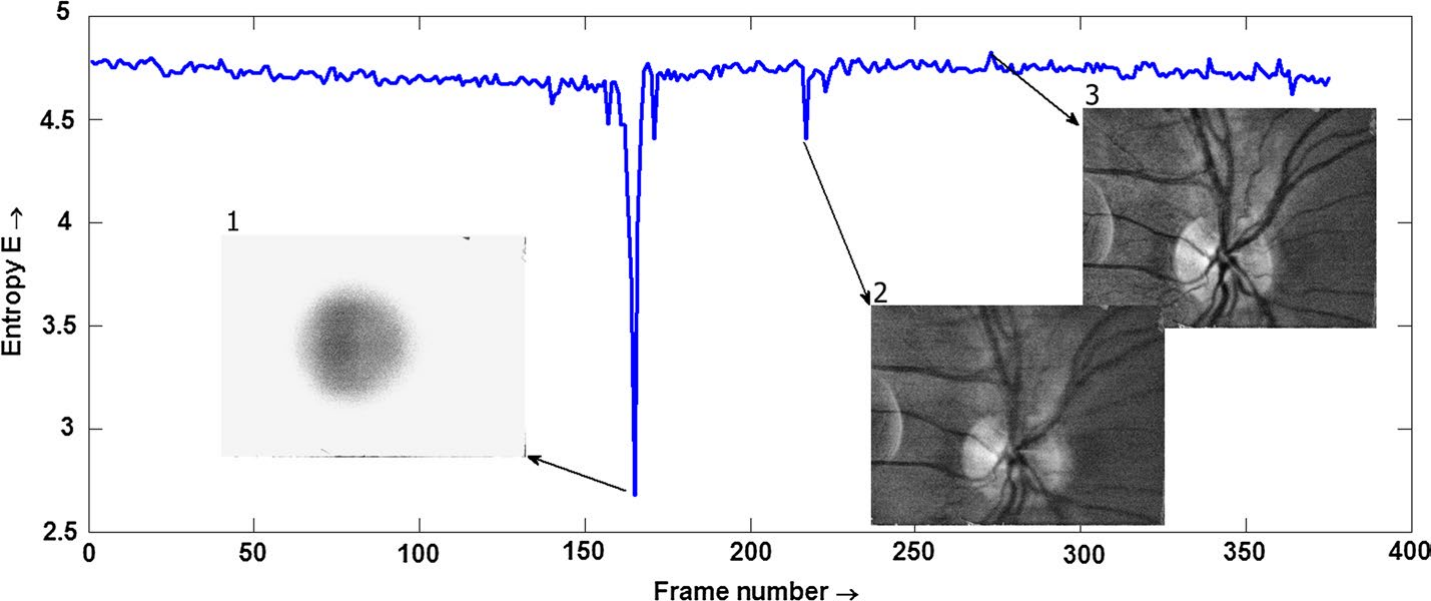
An example of entropy for one sequence with example of distorted frame due to eye blink (*1)*, eye movement (*2*), and the frame with the highest entropy value (*3*)

Here we omit the spatial variables *x*,*y* for simplicity. First, we consider that the low PSF distortion, i.e. *h*(*x*,*y*), will be similar to the Dirac function. The edge image (f*s) will be less blurred (the details in the image will be preserved) and the corresponding histogram (of the edge image) will have wider tonal distribution due to the presence of texture and noise. When the frame becomes blurred due to the time-variant or time-invariant part of PSF, the edge image will also be blurred, the details will smooth out, and the corresponding histogram will have sharper peaks. For very strong blurring, the edge image will contain similar intensity values, resulting in a peak in the histogram. If the frame *g*(*x*,*y*) contains high-intensity reflections (due to eye blinks or corneal reflection), the corresponding histogram will also contain one dominant sharp peak. Consequently, these histogram shape changes can be assessed by an entropy measure:

$$E =-\sum\limits_{i = 1}^{N} {p_{i} \log (p_{i} )}$$

where *p*_*i*_ is a probability density function estimated via histogram from the edge-enhanced image with *N* bins. The frames with low entropy will be distorted and the frame with the highest values can be taken as a reference frame. An example of entropy values for one sequence is shown in Fig. 2.

From an implementation point of view, the edge images have been obtained using the Sobel operator for horizontal and vertical directions, computed on a contrast-enhanced image (using contrast limited adaptive histogram equalization method—CLAHE [15]). The corresponding image histograms were computed with 128 bins.

### Sequence pre-processing

The recorded sequences are relatively noisy, mainly due to low-intensity retinal illumination. The signal-to-noise ratio (SNR), evaluated as a mean value to standard deviation in homogeneous region, is about 19 dB (estimated in several randomly selected frames and regions). In order to improve the efficiency of the registration method, we applied a simple median filtering with a 3 × 3 pixel window, which improves SNR up to 24 dB. The contrast of each frame was also equalised using the CLAHE method. This step increases the contrast of blood vessels, which is important for both steps of the registration process.

### Phase correlation

An FT-based (Fourier transform) technique of phase correlation can be used for alignment of two images. To create *rich* spectra, the images must have relatively high contrast and must contain edges. This approach can be used for estimating three spatial transformations and parameters—shift, rotation, and scaling. We use only shift, because the rotation is employed in the second stage of our registration approach and changes in scaling are not expected in our short term sequences. Let *g*_*1*_*(x,y)* and *g*_*2*_*(x,y)* be two frames, which differ only by displacements *x*_0_ and *y*_0_:

$$g_{2} \left( {x,y} \right) = g_{1} \left( {x - x_{0} ,y - y_{0} } \right)$$

which is equivalent to the following expression in spectral domain:

$$F_{2} \left( {u,v} \right) = F_{1} \left( {u,v} \right) \times e^{{ - j\left( {u \times x_{0} + v \times y_{0} } \right)}}$$

Taking the inverse FT of normalised cross spectrum leads to Dirac function at *(x*_*0*_*, y*_*0*_*)* position:

$$\delta \left( {x - x_{0} ,y - y_{0} } \right) = FT^{ - 1} \left\{ {\frac{{F_{2} \left( {u,v} \right) \cdot F_{1}^{*} \left( {u,v} \right)}}{{\left| {F_{2} \left( {u,v} \right) \cdot F_{1}^{*} \left( {u,v} \right)} \right|}}} \right\}$$

**Fig. 3:**
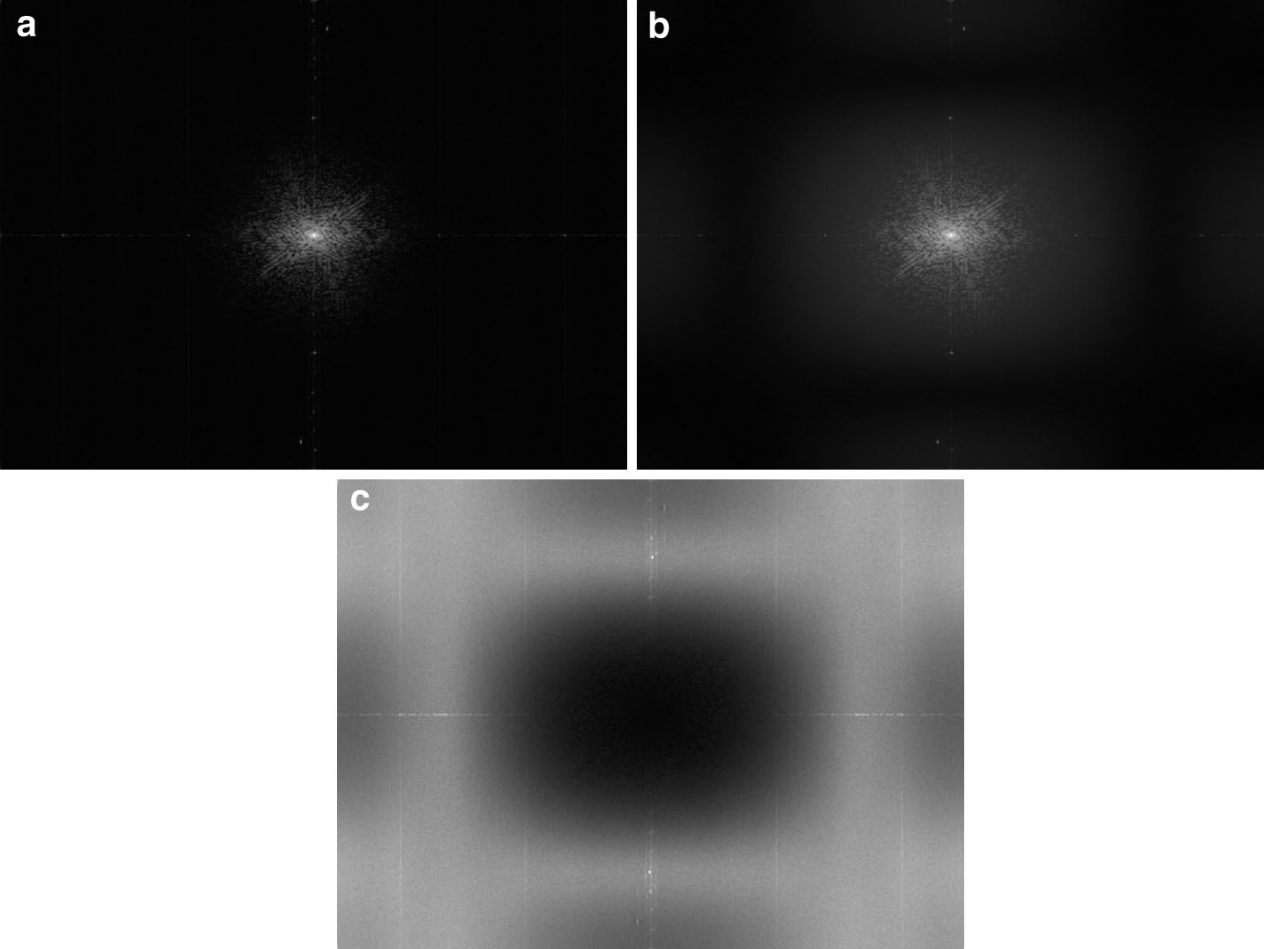
Images **a**, **b** show averaged spectra computed from one whole sequence: **a** spectrum from original sequence and **b** spectrum from 3 × 3 pixel median-filtered sequence (frame-by-frame). Image **c** shows spectral difference—the *dark* values in the middle represent “no change” around low frequencies, and the grey levels at higher frequencies represent reduced spectral components in median-filtered frames

In practical implementation the FT is replaced by DFT and a 2D window function has to be applied before taking this transform due to periodicity of DFT. A separable Hanning window with the size equal to the image was applied on each frame. Furthermore, as the Dirac function degrades to a sharp peak, its position must be detected in order to estimate *x*_0_ and *y*_0_ (using simple MAX operation, in our case). It must also be noted that image noise (typically at higher frequencies) can decrease the precision of shift estimation. As mentioned above, we applied a 3 × 3 pixel median filter on input images in the pre-processing stage, which suppresses the noise and preserves the edges (e.g. of blood vessels). This corresponds to the application of a low-pass filter window in the frequency domain, which is illustrated in Fig. 3 (see figure description for detailed information).

### Optical flow

**Fig. 4:**
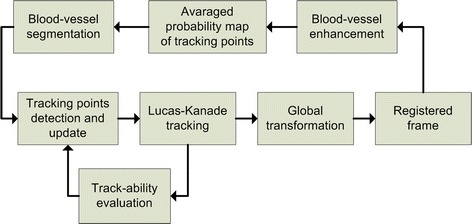
The basic scheme of the fine registration stage

Here we describe our approach to fine alignment of particular frames, which differs from our previously published work [14, 16], in several steps. The new concept is based on the gradual update of tracking points (TP) using information from the blood vessels and from the tracking stage. The basic scheme is shown in Fig. 4.

**Fig. 5:**
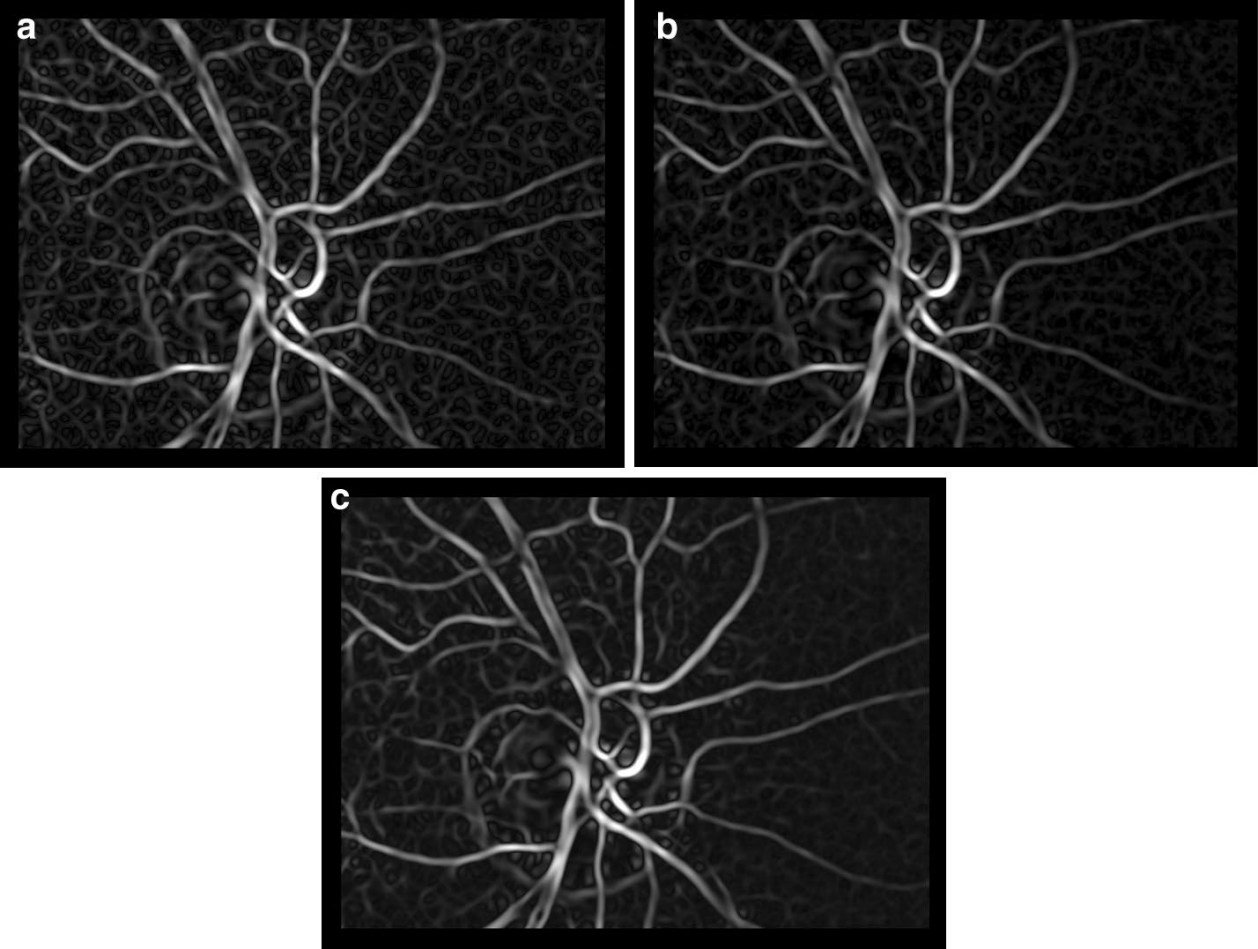
Tracking point probability maps for three different frames in the same sequence: **a** $\bar{\lambda }_{1} \left( {x,y} \right)$, **b** $\bar{\lambda }_{5} \left( {x,y} \right)$, **c** $\bar{\lambda }_{10} \left( {x,y} \right)$

We will start with TP detection, which is the most important part of the LK tracking approach. These points must lie on the retina and therefore we focused on the segmentation of blood vessels. A plethora of different approaches for blood-vessel segmentation have been published during the last three decades—see, for example, some recent papers [17, 18], or survey [19]. Here we employ a relatively simple and efficient method using the Hessian matrix, see [20]. This matrix is composed of the second derivatives computed in each pixel of the image—the Sobel difference operator approximates the derivatives. Since the blood vessels are darker than an image background (a valley), we can use higher positive eigenvalue from the Hessian matrix as a parameter to create a new eigenvalue image Λ(x,y). This image can be considered as a probability map of TP after appropriate normalisation as:

$$\lambda (x,y) = \frac{{\Lambda (x,y)-\Lambda_{\min}}}{{\Lambda_{\max} - \Lambda_{\min}}}$$

where Λ_min_ and Λ_max_ is minimum and maximum from eigenvalue image Λ(x,y), respectively. An example of this map is shown in Fig. 5, left.

In order to increase the precision of TP detection, taking into account possible temporal change of TPs, we are using the currently registered frame for modification of this probability map by means of image averaging with exponential weights:

$$\bar{\lambda}_{i} \left({x,y} \right) = q \cdot \bar{\lambda}_{i - 1} \left({x,y} \right) + \lambda_{i} \left({x,y} \right)$$

where 0 < *q*<1 weights the previously weighted eigenvalue image with respect to currently added image *λ*_*i*_*(x,y)*, starting with $\bar{\lambda}_{i} \left({x,y} \right) = \lambda_{1} \left({x,y} \right)$. The selection of *q* value is a trade-off between the influence of probability map from the current frame *λ*_*i*_*(x,y)* (high for low values of *q*) and influence of the exponentially weighted probability maps $\bar{\lambda}_{i} \left({x,y} \right)$ (high for high values of *q*). It can be deduced from our sequences that the majority of frames are acquired without distortion and only a few frames are distorted. Therefore, we chose a value close to 1 to decrease the influence of the current frame on the tracking-point selection. The normalisation according to the above-mentioned equation is performed after modification of each map. This averaging suppresses the random part while preserving the static part in the image, which can be seen from Fig. 5, where maps for *i* = 1, 5 and 10 are shown. This TP probability map is further modified using results from the LK tracking stage.

In our approach, we use the basic version of the Lucas-Kanade tracker [21]. Here, we briefly summarise this method and show how we use it for TP selection. The task of this tracker is to find, for a given tracking point *j* at position $X^{j}_{r} = \left({x^{j}_{r},y^{j}_{r}} \right)$ in a reference frame *g*_*r*_, corresponding TP $X_{i}^{j} = \left( {x_{i}^{j} ,y_{i}^{j} } \right) = X_{r}^{j} + D_{i}^{j}$ in frame *g*_*i*_, using the assumption that the neighbourhood of particular point $X_{r}^{j}$ is similar to the neighbourhood of point $X^{j}_{i}$ in defined rectangular window *W* of size *M* × *M*. The window size determines the trade-off between accuracy and robustness of the tracking. A smaller window leads to capturing small motion, but the tracking can be lost when the movement exceeds the size of this window. Conversely, a large window size can decrease accuracy. The large movements (up to a few pixels, see Table 1) have been compensated for by the phase-correlation approach and therefore the *M* value should be small. We set the window size at 31 × 31 pixels, which corresponds to the thickest blood vessels (about 21 pixels) and possible inter-frame movement in the range of a few pixels in our retinal scene.

**Table 1:** AME values and percentage evaluation of proposed method and combination of SIFT + LK tracking

	Proposed method Median ± st.dev. [px]	Phase-correlation part only Median ± st.dev. [px]	SIFT + LK tracking Median ± st.dev. [px]
Inside the ONH	0.78 ± 0.67	0.92 ± 0.78	0.82 ± 0.87
Outside the ONH	1.39 ± 0.63	1.62 ± 0.72	1.52 ± 0.73
# values <1 px (%)	33	28	30
# values between 1 and 2 px (%)	50	49	47
# values >2 px (%)	17	23	23

The displacement vector $D^{j}_{i} = \left({{\text{d}}x^{j}_{\text{i}},{\text{d}}y^{j}_{i}} \right)$ is referred to as the optical flow at $X_{i}^{j}$ and can take different values (e.g. size and direction) for each TP, because of the noise and possible non-rigid motion of retinal tissue. The displacement vector between one TP in frames *g*_*r*_ and *g*_*i*_ can be computed by minimising the mean squared error *ε*, which can be formalised as:

$$arg\left( {\mathop {\hbox{min} }\limits_{dx,dy} \varepsilon } \right) = arg\left( {\mathop {\hbox{min} }\limits_{dx,dy} \mathop \sum \limits_{{\left( {x,y} \right) \in W}} \left[ {g_{i} \left( {x + dx, y + dy} \right) - g_{r} \left( {x,y} \right)} \right]^{2} } \right)$$

Using Taylor series of the first-order on *g*_*i*_ and some trivial manipulation leads to (we omit the spatial indexes):

$$\varepsilon = \mathop \sum \nolimits \left[ {D_{i} + I_{x} \cdot dx + I_{y} \cdot dy} \right]^{2}$$

where *D*_*i*_ represents the temporal differences *g*_*i* *−*_ *g*_*r*_ and *I*_*x*_*, I*_*y*_ represents image of spatial horizontal and vertical differences of image *g*_*i*_, respectively. Taking derivatives according to unknown spatial shifts leads to a set of linear equations, which can be easily solved:

$$\frac{\partial \varepsilon}{\partial (dx)} = \sum {\left[{D_{i} + I_{x} \cdot dx + I_{y} \cdot dy} \right]} I_{x} = 0$$

$$\frac{\partial \varepsilon}{\partial (dy)} = \sum {\left[{D_{i} + I_{x} \cdot dx + I_{y} \cdot dy} \right]} I_{y} = 0$$

These two equations are applied for each TP (for each *j*) to get the optical flow $D^{j}_{i} = \left({{\text{d}}x^{j}_{i},{\text{d}}y^{j}_{i}} \right)$

Detection of reliable tracking points is the most important part of the LK tracker. After $dx_{i}^{j}$ and $dy_{i}^{j}$ are computed for each TP, we can use the same equation to compute the real value of these two summations, which can be considered as a measure of tracking-point quality (i.e. ‘trackability’) for each tracking point at position (*x*,*y*):

$$Q = \frac{1}{{2 \cdot M^{2}}}\sum\limits_{(x,y) \in W} {\left[{D_{i} + I_{x} \cdot dx + I_{y} \cdot dy} \right]} (I_{x} + I_{y})$$

The factor *M*^*2*^ normalises the value with respect to window size (*M* × *M*), because the sum is performed within this window and for *x* and *y* direction (factor two for two windows).

**Fig. 6:**
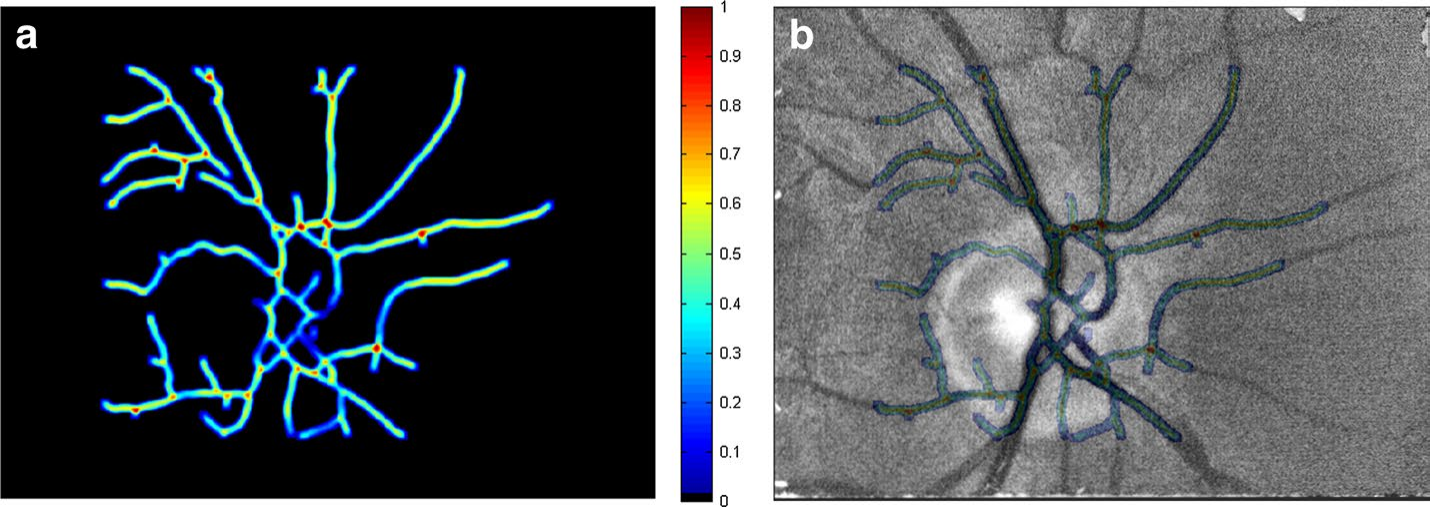
An example of TP quality image (*left*) and an overlay with current frame

Figure 6 shows one *Q*_*i*_*(x,y)* image for selected frame *i* during the registration process after normalisation to (0, 1) range, where 1 represents good TP and 0 unreliable TP. One can see that the TPs with low error are placed at the blood-vessel bifurcations or crossings. It should be noted that the original *Q*_*i*_(*x*,*y*) image has been convolved with a Gaussian kernel, which spatially spreads the TP quality value in order to estimate the TP quality in neighbourhood pixels—because the tracking is not based on a single pixel but a pre-defined window: *W*.

This TP quality map $Q_{i} \left({x,y} \right)$ is consequently used to modify the selection of the TPs for the next frame using currently computed TP probability map $\bar{\lambda }_{i} \left( {x,y} \right)$:

$$\bar{\lambda }_{i+1}( {x,y}) = \bar{\lambda }_{i} ( {x,y})\cdot( {1 + Q_{i} ( {x,y})})$$

The TP probability map is linearly increased only at such locations for which the map is higher than zero.

This modified eigenvalue image $\bar{\lambda }_{i} \left( {x,y} \right)$ is further thresholded to get the candidates for tracking points for the next frame. The threshold is estimated using the Kittler optimal thresholding approach [22]. Small, segmented regions are consequently removed as we assume that segmented blood vessels should create longer (i.e. larger) structures. Skeletisation is finally used to obtain the vessel centrelines, which are used for LK tracking with updated TPs.

The global transformation is then applied to register each frame to the reference frame. We used shift and rotation transformation for particular frame alignment. Shift and rotation parameters of this transformation are estimated by solving an overestimated set of *N* equations (for *N* tracking points), minimising the following expression:

$$\sum\nolimits_{J = 1}^{N} {\left| {\left({\begin{array}{*{20}c} {x_{j}^{ref}} \\ {y_{j}^{ref}} \\ \end{array}} \right) - \left({\begin{array}{*{20}c} {x^{\prime}_{j}} \\ {y^{\prime}_{j}} \\ \end{array}} \right)} \right|}^{2} \to \min,$$

where $$\left({\begin{array}{*{20}c} {x^{\prime}_{j}} \\ {y^{\prime}_{j}} \\ \end{array}} \right)$$ are the coordinates of tracking points after the Euclidian transformation:

$$\left({\begin{array}{*{20}c} {x^{\prime}_{j}} \\ {y^{\prime}_{j}} \\ \end{array}} \right) = \left({\begin{array}{*{20}c} {\cos (\varphi)} & {- \sin (\varphi)} \\ {\sin (\varphi)} & {\cos (\varphi)} \\ \end{array}} \right) \times \left({\begin{array}{*{20}c} {x_{j}} \\ {y_{j}} \\ \end{array}} \right) + \left({\begin{array}{*{20}c} {tx} \\ {ty} \\ \end{array}} \right)$$

and the $$\left({\begin{array}{*{20}c} {x_{j}^{ref}} \\ {y_{j}^{ref}} \\ \end{array}} \right)$$ are coordinates of tracking points in the reference image. The geometrical parameters are finally found using the Gauss elimination method.

The process of LK tracking and global transformation is iteratively applied until the change of the geometrical parameters (*tx, ty* and $\varphi$) is small—between two iterations. The threshold is set to 0.05 pixels for shifts and 2 degrees for rotation. The typical number of iterations is between 2 and 7 for the whole data set.

## Results

### Evaluation

**Fig. 7:**
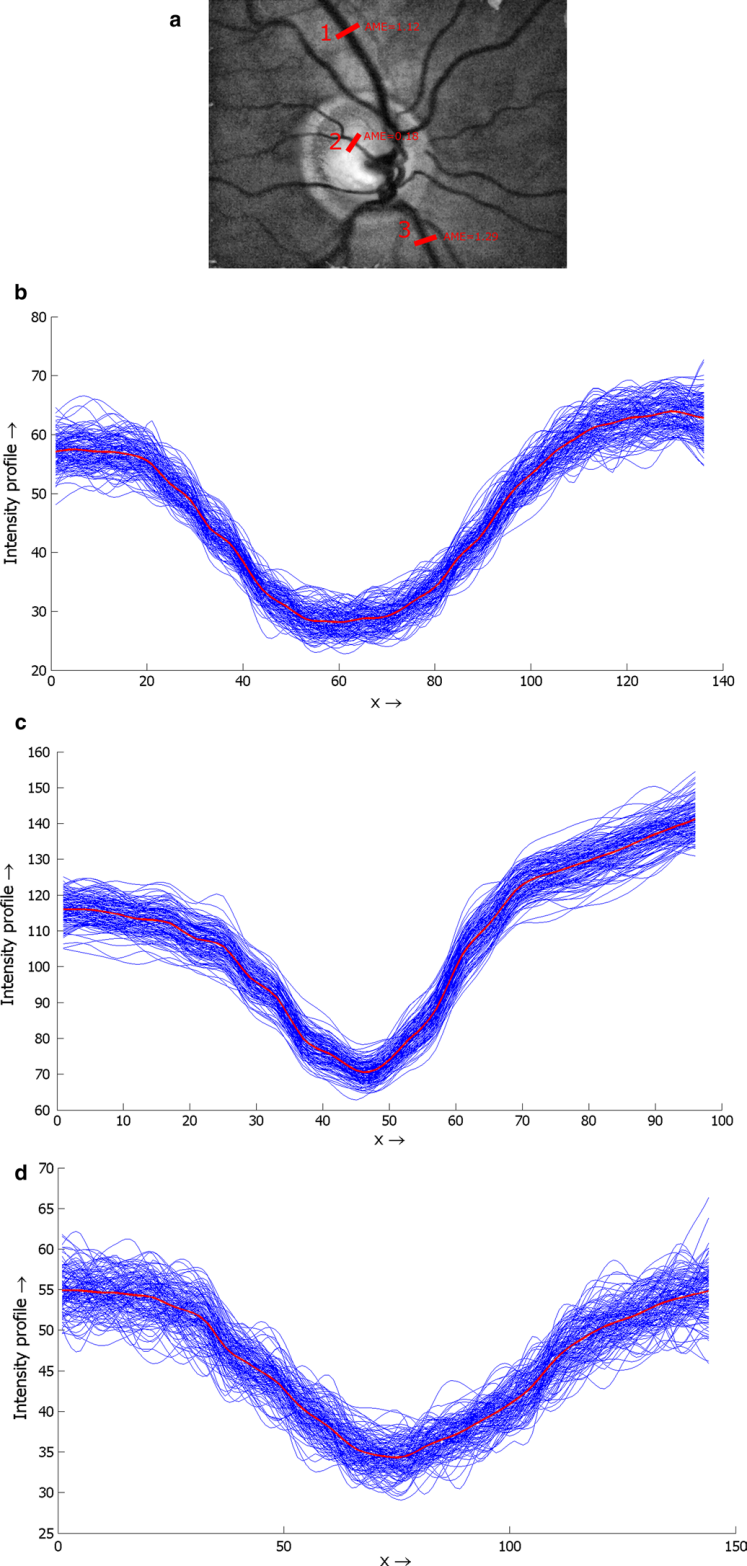
An example of the line profile selection (**a**) and real profile curves corresponding with position *1* (**b**), *2* (**c**) and *3* (**d**), together with the mean curve (*red*)

The evaluation of the method is also based on retinal vasculature. The blood vessels are stable and visible structures on the retina and shouldn’t move in registered sequence, except for the diameter changes. The pulsations are visible in the main retinal arteries but can be observed also as spontaneous venous pulsations [23]. Nevertheless, we assume that the centre of the blood vessel should remain at the same position. We therefore proposed that the evaluation algorithm be based on constancy of the blood-vessel centrelines, as follows:

1. Manually choose positions for evaluation on the vascular tree.
2. Compute the mean blood-vessel intensity profile IP_mean_ from the whole sequence (Fig. 6). Each frame is filtered with a 3 × 3 pixel averaging filter before this extraction in order to slightly suppress the noise and get smoother intensity profiles.
3. Upsample IP_mean_ with factor 4 and determine the position of minimum value X_mean_. The upsampling *artificially* increases spatial resolution and better approximates the position of the intensity-profile minima.
4. For each intensity profile IP_i_ (extracted from each frame, *i*) determine its minimum value X_i_ and determine the absolute mean error (AME) for whole sequence:

$${{AME}} = \frac{1}{4 N}\sum\limits_{i = 1}^{N} {\left| {X_{i} - X_{mean}} \right|},$$

where *N* is the number of frames in the sequence and number 4 corresponds to the upsampling factor. These steps are repeated for different positions selected in the first step. This evaluation approach computes the mean differences from the most probable position of the blood vessels centred at specific places. Therefore, it gives a measure of registration precision, although there can be higher individual AME values. We used 18 positions, which were determined manually in different locations in a reference frame (see Fig. 7 for illustrative example).

We have tested the precision of the registration in two regions separately—inside and outside the ONH. This is because the contrast of blood vessels is usually higher inside the ONH region, as the ONH is brighter than the surrounding retinal tissue. Therefore, we need to assess whether the precision is influenced by this image/sequence property, because both stages of the registration process utilise retinal vascularity.

For performance comparison, we’ve used a basic and often-used approach combining the SIFT detector and LK tracker. Basic LK tracking has been applied on sequences after phase-correlation alignment. Vedaldi’s Matlab implementation of the SIFT method has been used [24]. The results are summarised in Table 1. We evaluated only the results of the phase-correlation part.

**Fig. 8:**
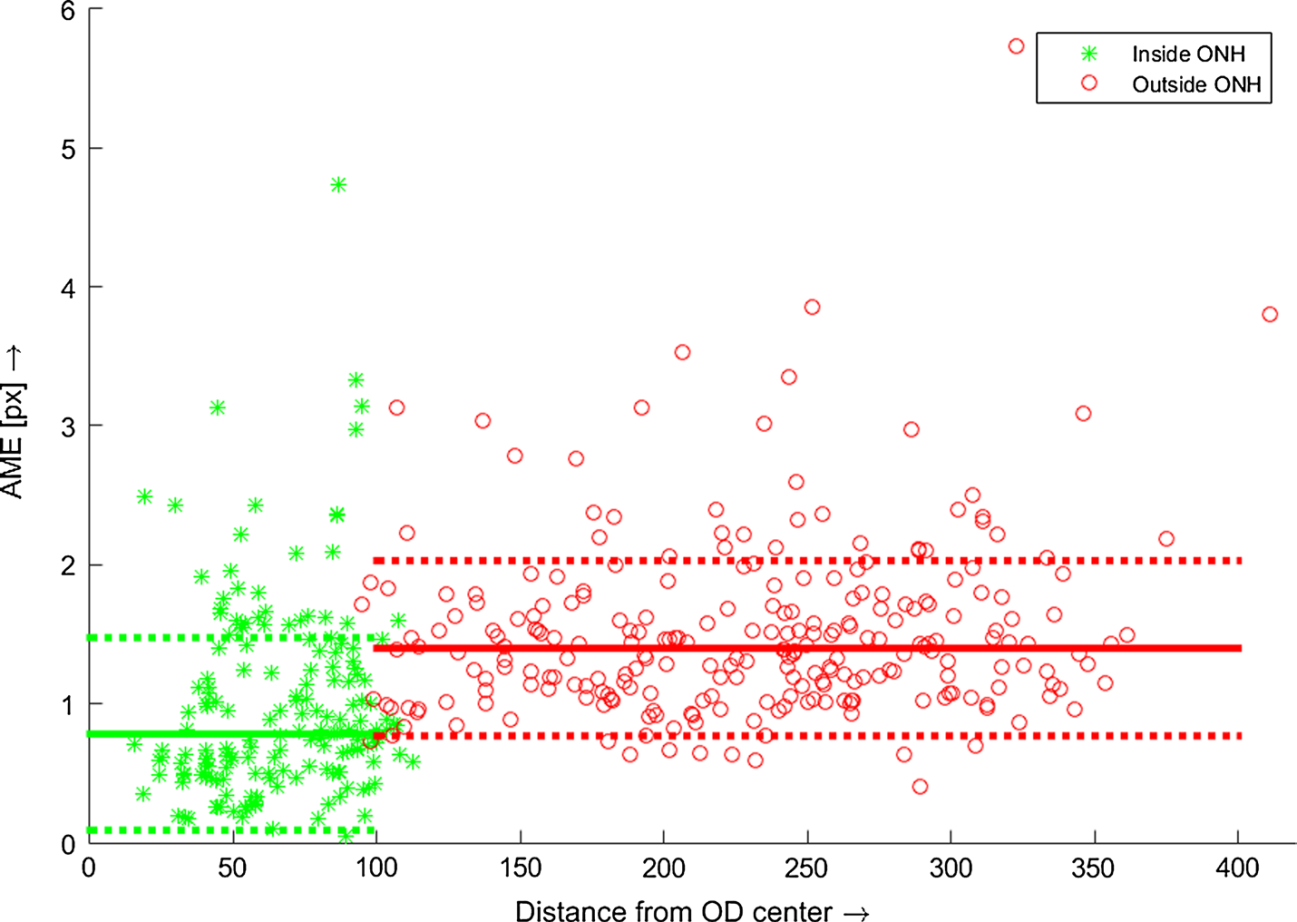
AME values for all sequences, computed on manually selected blood vessel cross-sections and plotted in *different shapes* and *colours* for positions *inside* and *outside* the ONH. The *full lines* represent median values for each set separately and the *dotted line* represent standard deviation

The ONH position, approximated by a circle, has been manually determined in each registered sequence. Then the evaluation has been performed within both regions separately. The plot in Fig. 8 shows a relation between the AME value and the distance from the ONH centre for all sequences. The green stars correspond to the inside of the ONH and the red circles to the outside. We applied the Mann–Whitney U test to test the hypothesis that the AME values from both regions have equal medians. The null hypothesis tests the same medians that have been rejected, showing that the difference is statistically significant at 1 % significance level. This finding is important, because many retinal image registration approaches rely on segmented blood vessels. Nevertheless, this difference is only 0.61 px, which is below one pixel, showing that the error difference is relatively small and can be safely neglected in most applications. The standard deviations of AME errors from both regions are the same (tested by two-sample F test). The difference in median values can be clearly seen in Fig. 8. One important remark must be stressed here. The higher AME value is influenced not only by the registration method, but also by the image quality and the quality of the blood-vessel intensity profile, respectively. It can be seen in Fig. 7 that the intensity profiles extracted from vascularity outside the ONH are generally noisier and have lower contrast in comparison with the profiles extracted from the region inside the ONH. This probably results in slightly higher AME values.

We also explored in more detail the situations for higher AME values. In some sequences, there are several frames that contain the above-mentioned artefacts (motion blur, reflection). These frames were detected using the frame entropy and appropriate threshold (adjusted manually), thus removing them from evaluation. Nevertheless, some less-distorted frames (e.g. frames with small corneal reflections or frames with lower signal-to-noise ratio) are still presented even after this processing, which might influence the AME values.

The standard approach using the SIFT + LK method achieved slightly higher median values as well as higher variances—see Table 1. Furthermore, the standard deviations for both regions significantly differed (tested by two-sample F test) and both values were higher than standard deviations for the proposed approach.

We also evaluated the amount of AME values in different ranges for both approaches (Table 1). We found that over 80 % of evaluated blood vessel cross-sections were registered with an absolute mean error of less than 2 pixels, while the SIFT + LK approach achieved less than 80 %.

**Fig. 9:**
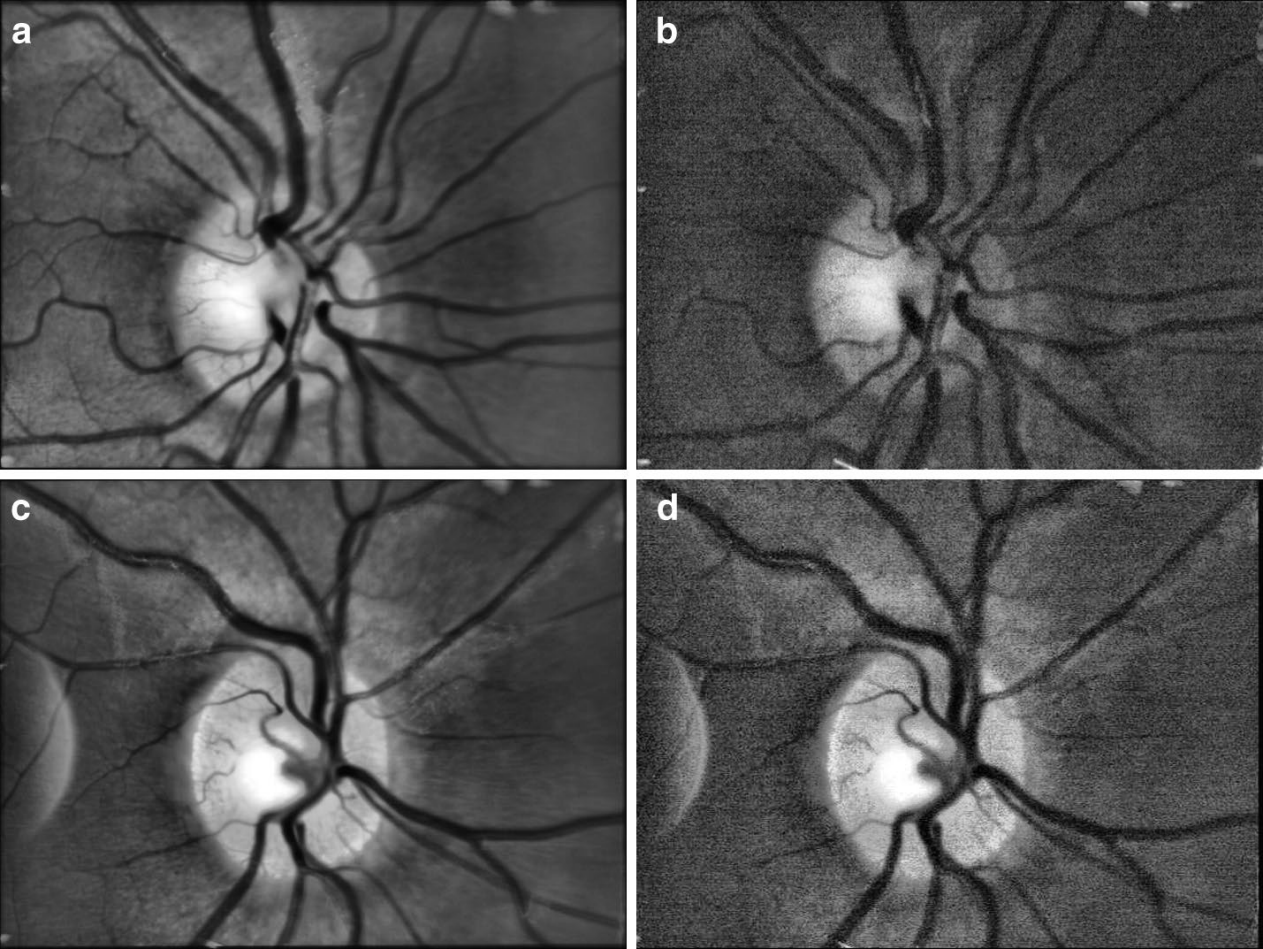
Two examples of averaged retinal images from two sequences (**a**, **c**) and single frame from corresponding sequence (**b**, **d**). The CLAHE method has been applied on each image to increase the contrast

Finally, we show two examples of an averaged retinal image computed from a corresponding sequence after an adaptive histogram equalisation (Fig. 9). The comparison with a single frame is also shown.

### Implementation and computational complexity

The CPU processing time has been measured by the *cputime* Matlab function. The hardware used for testing comprises Intel^®^ Core™ i7-4710MQ, running at 2.5 GHz, and 16 MB RAM. The average values per frame processing are shown in Table 2. The values are based on Matlab built-in and developed functions, without any additional *C* implementation. As Matlab uses high-level “scripting” language, the source code is translated line-by-line into machine instructions, which decreases the execution speed. Therefore, the processing of the whole sequence takes typically 1–2 min in current implementation. The implementation in some low-level language typically provides much higher computational speed (at levels of 1 to 2 orders faster), which can result in a computation time in the range of a few seconds. There are also efficient implementations of LK tracker, which are used for real-time implementation on Full HD video, e.g. in Mahmoudi et al. [25]. The increase of computation time when using the proposed approach instead of the SIFT + LK approach is about 30 %, which is acceptable in cases of more efficient implementation.

**Table 2:** Averaged CPU time values per one frame processing as measured in Matlab

	Entropy calculation (s)	Phase correlation, stage 1 (s)	LK tracking, stage 2 (s)
Proposed method	0.22	0.25	2.5
SIFT + LK	0.22	0.25	1.9

## Conclusion

We described an advanced image-processing method for offline registration of video sequences acquired by an experimental, low-cost video-ophthalmoscope. We showed that the application of a phase-correlation method together with LK tracking and “frame-to-frame” modification of the tracking point could successfully be used for this task. We also described an evaluation approach that can be used if there is no “gold-standard” dataset, which is frequently the case in the video-processing area. Our evaluation showed that the registration method could achieve acceptable results for most of the possible applications. These applications cover, for example, evaluation of blood-vessel pulsation [26], eye-movement estimation [16], and super-resolution using multiple frames [27].

The experimental video-ophthalmoscopic camera has a flexible design and can be relatively easily modified for some specific applications. For example, the laser speckle contrast imaging would need to include a coherent light source instead of simple LED; the application for monitoring fast-movement changes would need a faster camera with higher frame rate; and using a brighter broadband light source would provide a lightweight and easy-to-use colour camera. Nevertheless, all these applications would need a reliable method for registration of acquired sequences.
